# Developing infographics to implement patient blood management in the surgical pathway: From concept to design

**DOI:** 10.1016/j.htct.2026.106473

**Published:** 2026-05-30

**Authors:** Matheus Santos Moitinho, Bianca Meneghini, Rosangela Monteiro, Anderson Manucci, Maria Carolina Guido, Guilherme Rabello, Fabio Biscegli Jatene

**Affiliations:** a Núcleo de Inovação (InovaInCor), Instituto do Coração, Hospital das Clínicas HCFMUSP, Faculdade de Medicina, Universidade de São Paulo, São Paulo, Brazil; b Divisão de Cirurgia Cardiovascular, Instituto do Coração, Hospital das Clínicas HCFMUSP, Faculdade de Medicina, Universidade de São Paulo, São Paulo, Brazil

**Keywords:** Health communication, Health education, Blood transfusion, Blood conservation, Patient care management

## Abstract

**Introduction:**

Effective communication plays a crucial role in health education by facilitating the dissemination and assimilation of complex clinical concepts. Infographics are promising visual tools to promote evidence-based practices, especially for multifaceted strategies like patient blood management.

**Objective:**

The aim of this study was to develop and evaluate infographics to communicate Patient Blood Management principles along the surgical pathway.

**Methods:**

Using a user-centered, iterative Design Thinking approach, a multidisciplinary team of healthcare professionals, a graphic designer, and patient blood management specialists developed infographics across five stages: narrative construction, sketching, digital design, expert validation, and assessment of real-world applicability.

**Results:**

Infographics representing patient blood management pillars were created incorporating icons, color gradients, symbolic pathways, and QR codes linking to supplementary content. The materials were accessible, visually engaging, and technically accurate, facilitating knowledge retention and practical application of patient blood management protocols.

**Conclusion:**

Infographics can overcome educational barriers and enhance the engagement of healthcare professionals in patient blood management implementation. By combining scientific evidence with visual communication, infographics support cultural shifts towards rational blood use, aligning with global surgical safety and public health objectives.

## Introduction

Communication, whether visual, written, or verbal, is a fundamental pillar for the effective dissemination of knowledge. The way information is conveyed, through images, texts, or speech, directly shapes individuals’ perception, assimilation, and engagement with the presented content [1]. In this context, infographics represent a powerful fusion of visual and written communication, structuring complex information into clear and accessible representations [2,3].

The human brain processes images much faster than isolated texts, and the simultaneous combination of visual and textual stimuli activates multiple brain areas related to memory and learning [4,5]. This integration facilitates understanding and retention of knowledge, translating complex information into more easily assimilated concepts and promoting greater engagement and practical application [6].

In healthcare, where the volume of technical information is high and time for assimilation can be limited, innovation in communication plays an even more strategic role. Infographics emerge as innovative tools to translate scientific data and clinical protocols into visually appealing and educational formats, aiding continuous education and adherence to evidence-based practices [7].

To validate this context, the concept of Patient Blood Management (PBM) was chosen as the subject for applying infographics. PBM, recognized by the World Health Organization (WHO) as a patient-centered, systematic, evidence-based approach to improve patient outcomes by managing and preserving a patient’s own blood, while promoting patient safety and empowerment, encompasses three main pillars: management of preoperative anemia, minimization of intraoperative blood loss, and optimization of postoperative care [8, 9, 10, 11].

Given the importance of PBM for patient safety and healthcare sustainability, infographics are a powerful tool to overcome cultural barriers, raising awareness and engaging healthcare professionals. Thus, this study describes the development of these visual materials, highlighting their creation, design strategies, and potential impact on public health education, underscoring the transformative role of visual communication in modernizing and enhancing the dissemination of clinical knowledge.

## Methods

### General graphic design framework

The development of the infographics followed an iterative creation approach focused on the end-user’s information absorption. It is based on the principles of Design Thinking [12] for the development of graphic elements. The adopted approach placed the content-consuming professional as the main focus in the development process, emphasizing continuous iterations, collective feedback, and careful consideration of the context in which the innovation was implemented.

### Development team

The infographic development process was conducted by a multidisciplinary team composed of professionals including a nurse, a biomedical scientist, biologists, a graphic designer, and an engineer. Some members of this team are PBM specialists (certified by the Sociedad Iberoamericana de PBM [SIAPBM] or the Society for the Advancement of Patient Blood Management [SABM]).

### User-centered development stages

**Fig. 1:**
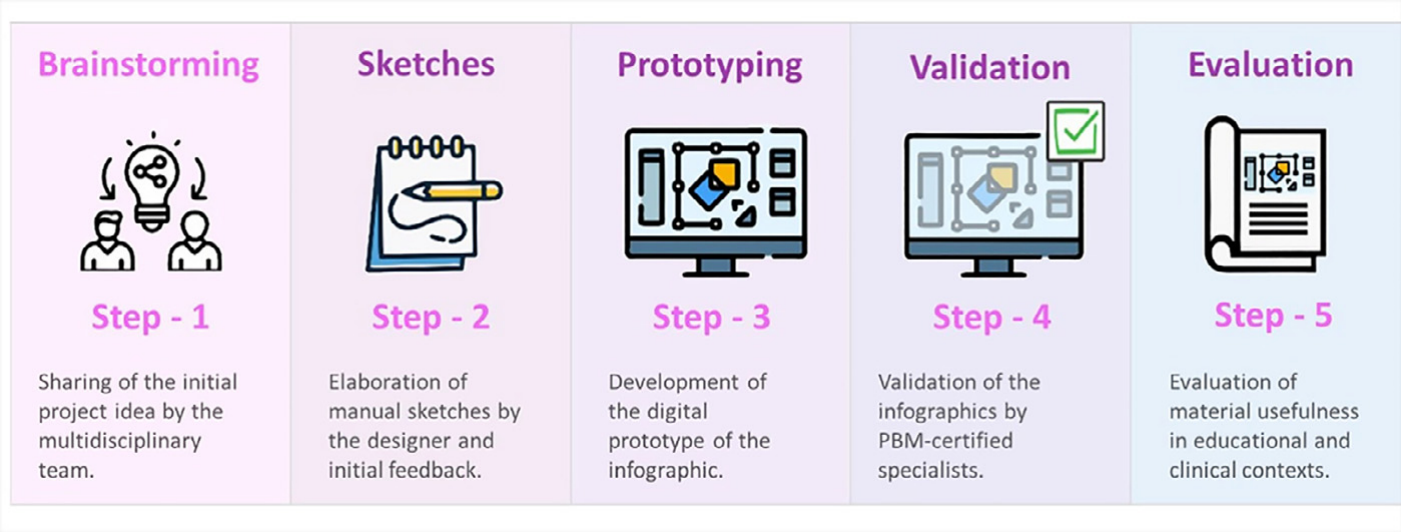
Illustration of the infographic development stages.

For the creation of the infographics, five structured development stages were carried out to explore specific aspects, using techniques such as storytelling [13], brainstorming for graphic elements, and feedback iterations of the surgical patient journey (Figure 1).

The first stage involved the multidisciplinary team sharing the initial project ideas. During this session, the team shared their practical experiences related to PBM and the challenges associated with understanding its principles. These narratives helped shape the initial infographic content, directly connecting it to practical, real-world clinical scenarios. Additionally, brainstorming was employed in this stage to generate creative ideas and explore innovative approaches to infographic construction. This exchange aimed to stimulate concrete reflections on clinical practice problems and explore possible educational solutions that could be translated into visual elements.

In the second stage, the first infographic prototypes were presented, developed as hand-drawn sketches by the design professional. These sketches served as basic, schematic representations of the infographics, allowing the team to explore content organization, visual hierarchy, and initial layout before moving on to detailed designs. The initial feedback on the sketches from the team was crucial to identify needs such as simplifying technical language, increasing the use of icons, and structural adjustments to facilitate the understanding of different audiences.

Based on the adjusted sketches, the third stage focused on developing digital prototypes. During this phase, graphic elements such as colors, diagrams, and figures were refined, prioritizing message clarity and visual impact. This iterative approach between sketches and prototypes ensured that changes reflected end-user needs and maintained consistency with established educational and design principles.

The fourth stage consisted of an analysis of the infographics by the PBM-certified experts. In this phase, the content was thoroughly reviewed to ensure technical accuracy and practical relevance.

Finally, the usefulness of the materials was validated in potential real educational and clinical contexts, allowing a final analysis of their applicability and effectiveness as educational tools.

The Design Thinking methodology [12] was present throughout all development stages, acting as the central axis for the conception, refinement, and validation of the graphic representations. This creative and iterative approach model enabled a structured construction of the materials, ensuring that the infographics were visually appealing, scientifically grounded, and didactically effective in disseminating PBM concepts. Moreover, the iterative and collaborative development process guaranteed that the infographics met the target audience’s expectations and contributed to the dissemination of PBM principles.

### Digital version of the infographic

The infographic development utilized CorelDRAW version 23 and Adobe Photoshop version 25.12.0. CorelDRAW was used to create illustrations, vector elements, and structured layouts, while Adobe Photoshop was employed for image editing and manipulation, ensuring visual refinement and the final quality.

## Results

### Infographic 1: the patient blood management of anemia in the ‘Surgery verse’

#### Conceptual description

This infographic simplifies a complex medical protocol using a gamified visual learning experience. It leverages a metaphor of a journey or a ‘verse’ to guide viewers through the PBM process for anemia before surgery.

The innovative approach is evident in how it visualizes the decision-making pathways. Patients are represented as figures on a path, creating a clear narrative flow. The initial ‘Check Control for Anemia’ acts as a critical gatekeeper. Instead of just listing criteria, the infographic shows the diverging paths: those who are cleared proceed directly to the goal (‘Surgery’), while those who are not are rerouted for intervention.

The detour is where the educational power truly shines. It graphically represents the diagnostic and treatment phases, a ‘blood analysis’ followed by ‘treatment’ with key supplements (iron, vitamin B12, Folic Acid, erythropoietin). The color-coded bar for treatment is a simple yet effective way to represent a spectrum of care. The concept of a ‘Detour Return’ highlights the iterative nature of PBM: it is not a one-and-done process. Patients are re-evaluated in a second ‘check for anemia’ before they can be cleared for surgery. This loop reinforces a core principle: patient optimization is paramount and may require multiple steps.

By personifying the patient's journey and using visual cues like green checkmarks for success and red 'X's for setbacks, the infographic transforms a dry medical guideline into a compelling, easy-to-understand narrative.

#### Visual elements

The dark background creates contrast and highlights the vibrant colors of technical figurative elements, making the visual flow clearer and more appealing. For the figurative elements, shades of blue, purple, and cyan are used to represent stages such as hematological analysis and medical guidance.

**Fig. 2:**
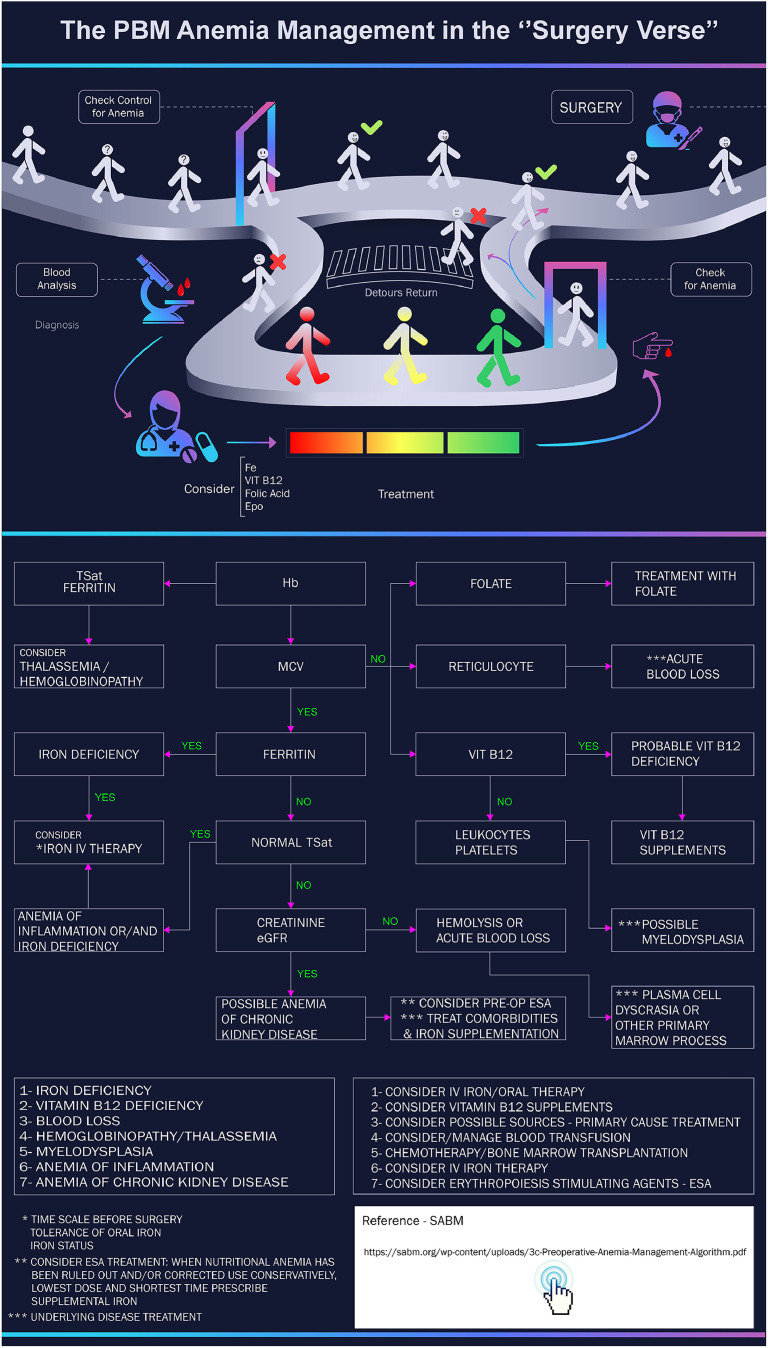
Patient blood management of anemia in the ‘Surgery Verse’. This gamified visual narrative guides viewers through preoperative anemia screening, diagnostic tests, and sequential treatments (iron, vitamin B12, folic acid, erythropoietin). The iterative re-assessment step ensures patient optimization before surgery. Stylized patient Fig.s, color-coded paths, and minimal text create an engaging and intuitive clinical decision flow. Accessible via QR code, this resource provides an in-depth diagnostic algorithm considering hemoglobin, ferritin, transferrin saturation, vitamin B12, and reticulocytes. It guides personalized treatment choices including oral/IV iron, vitamin supplementation, and erythropoietin use, supporting evidence-based clinical decisions. ESA: Erythropoiesis-stimulating agent.

The use of minimalist stylized human figures facilitates understanding of the patient’s trajectory. Iconography such as a microscope, doctor, and blood are stylized in a simple and clear manner. The patient’s path is represented by a continuous curved line, providing a sense of movement and continuity. Arrows indicate directions and actions. Text is minimal and direct, complementing the visuals. The use of symbols predominates to convey messages quickly (Figure 2).

Figure 2 Fe; Iron; VIT B12: Vitamin B12; EPO: Erythropoietin; TSat: Transferrin saturation; Hgb: Hemoglobin; MCV: Mean corpuscular volume; IV: Intravenous; ESA: Erythropoiesis stimulating agents.

#### QR codes and informative supplementation

As part of the additional content of Infographic 1, a QR code was incorporated, directing users to supplementary materials on preoperative anemia management. This includes a detailed flowchart guiding decision-making according to the type of anemia, covering everything from initial laboratory screening to defining the appropriate therapeutic approach. The image presents a structured scheme for differential diagnosis of anemia, considering parameters such as hemoglobin, ferritin, transferrin saturation, vitamin B12, and reticulocytes. Based on these data, the flowchart guides the healthcare professional in choosing treatment, including oral or intravenous iron supplementation, vitamin B12 or folate replacement, erythropoietin use, and other strategies according to the etiology of the anemia (Figure 2).

### Infographic 2: the patient blood management ‘Winner's lap’ in the ‘Surgery verse’

#### Conceptual description

This infographic cleverly transforms the medical interventions into a structured, metaphorical ‘winner's lap’, a concept familiar from racing, to underscore the goal of a successful, safe surgery.

The innovation lies in its use of a circular, continuous path to represent the perioperative process. It eschews a linear flowchart for a dynamic loop, emphasizing that PBM is not just a pre-surgical checklist but a continuous cycle of care. Patients, again represented as figures on a path, first encounter a ‘Pre-surgery check-point’ that includes obtaining informed consent and creating a PBM surgical plan. This step is presented as a crucial start line. The central part of the infographic is a visual tour of the operating room, where various PBM strategies are presented as numbered stations around a central operating table. This format effectively breaks down the intraoperative phase into a series of distinct, yet interconnected, interventions. This ‘pit stop’ style of presentation makes the various tools and techniques of PBM easy to digest and remember.

**Fig. 3:**
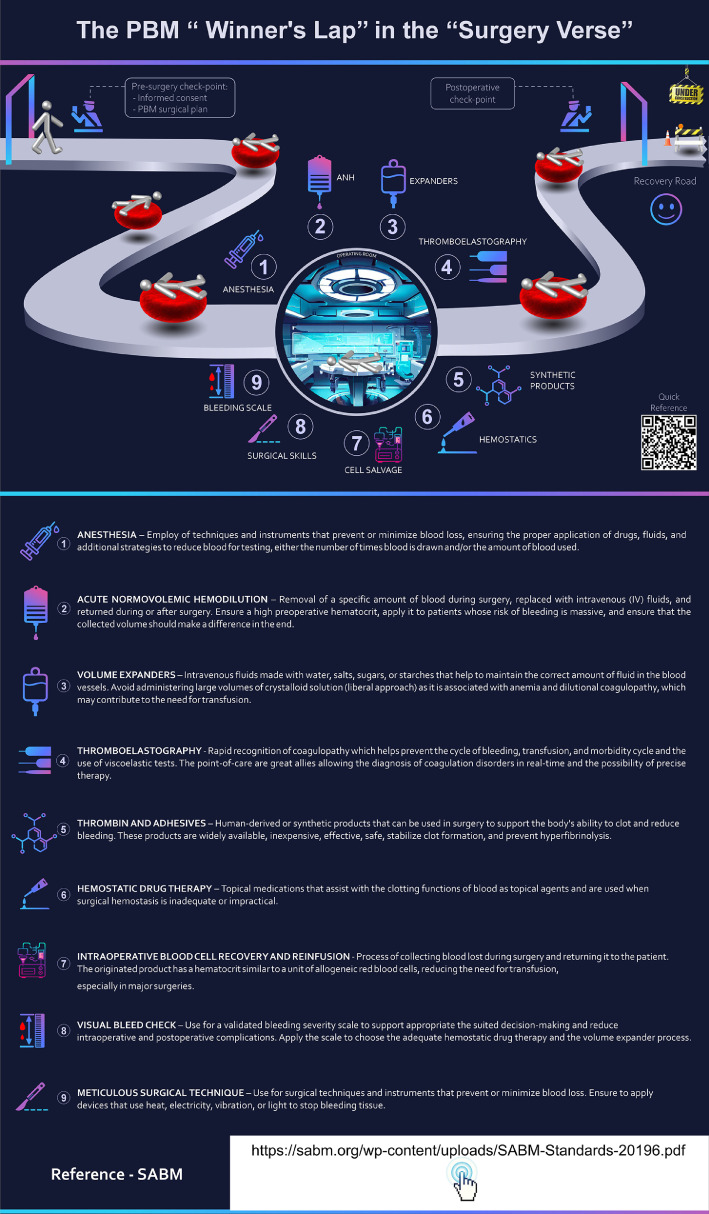
The patient blood management (PBM) ‘Winner’s Circle’ in the ‘Surgery Verse’. This infographic depicts PBM as a continuous, circular pathway encompassing preoperative planning, intraoperative interventions, and postoperative recovery. Patient Fig.s progress through numbered ‘pit stops’ around a central operating table, visually breaking down complex surgical blood management strategies into digestible steps. Linked via QR code, this guide summarizes each intraoperative PBM strategy with clear indications and usage guidelines, enhancing accessibility and supporting clinicians in applying best practices during surgery.

After the central ‘winner's lap’ of interventions, the patient figure moves towards a ‘Postoperative check-point’ and then onto the ‘Recovery Road’. This sequential layout reinforces the full spectrum of care, from pre-op planning to post-op recovery (Figure 3).

### Visual elements

As described in the Infographic 1, the background, the figurative elements and, the use of minimalist stylized human figures facilitates understanding of the patient’s trajectory.

Iconography and the patient’s path were chosen to represented a sense of movement and continuity. The use of symbols predominates to convey messages quickly (Figure 3).

### QR codes and informative supplementation

The inclusion of a QR code for a ‘Quick Reference’ further enhances its utility as a modern, accessible learning resource. This infographic describes all processes numbered in the surgical phase and the guidelines for each one (Figure 3).

### Infographic 3: the patient blood management ‘Energy wheel’ in the ‘Surgical verse’

#### Conceptual description

This infographic presents an innovative model for understanding PBM by reframing the process as a dynamic system. Instead of a linear path or a static checklist, it uses the metaphor of an ‘Energy Wheel’ to illustrate how various interventions work together to drive a patient towards a successful outcome.

The central spinning turbine visually represents the core engine of PBM, with each blade symbolizing a critical component. This design shows that all these elements are interdependent and must be activated in a coordinated manner for the system to function effectively. The wheel is surrounded by eight key PBM strategies, each with a corresponding icon presented as the fuel or power that turns the wheel.

**Fig. 4:**
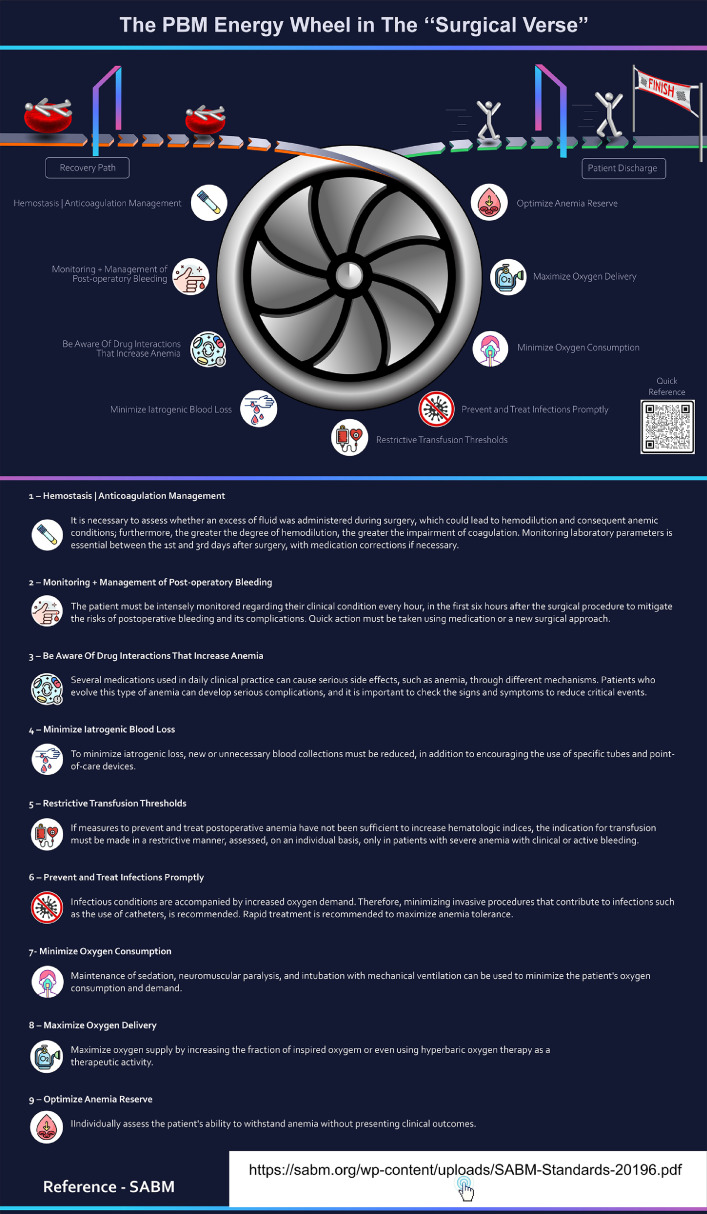
The patient blood management (PBM) ‘Energy Wheel’ in the ‘Surgery Verse’. This innovative model uses a spinning turbine metaphor to emphasize the interdependent PBM strategies powering the patient’s journey from intervention to recovery. Each blade represents a critical component, reinforcing the need for coordinated, continuous care to achieve optimal outcomes. Accessible through QR code, this content provides detailed recommendations on bleeding and thrombosis prevention, postoperative monitoring, and therapies to optimize oxygen delivery and consumption, reinforcing safe and effective blood management.

The patient's journey is shown as a path leading into and out of this central system. Patients are depicted entering the process, engaging with the ‘Energy Wheel,’ and then proceeding on the ‘Recovery Path’ toward ‘Patient Discharge’ and the ‘FINISH’ line. This graphical representation transforms a set of guidelines into an active, process-oriented model, highlighting that PBM is not a static state but a continuous, dynamic effort to power a patient's recovery (Figure 4).

#### Visual elements

As described in the Infographic 1, the background, the figurative elements and, the use of minimalist stylized human figures facilitates understanding of the patient’s trajectory.

Concise and direct texts associated with each icon ensure the message is quickly assimilated, while the overall composition reinforces clarity and organization of the information (Figure 4).

#### QR codes and informative supplementation

This interactive link provides access to detailed strategies for managing hemostasis and anticoagulation, addressing ways to prevent both bleeding and thrombotic complications, as well as offering recommendations for rigorous monitoring of postoperative bleeding. In addition, the QR code directs users to therapeutic approaches for different types of anemia, with content on strategies to optimize oxygen delivery and consumption, highlighting interventions that ensure adequate tissue perfusion and minimize metabolic demand in anemic patients (Figure 4).

## Discussion

This study demonstrates the potential of infographics as effective tools to communicate and disseminate the principles of PBM across surgical care. The visual materials developed present clinical pathways and protocols in a structured, intuitive format that enhances understanding and engagement, particularly within multidisciplinary healthcare teams.

The choice of PBM as the central theme is justified by its growing relevance in the global health landscape, particularly following recommendations from the WHO, which recognizes this approach as essential for patient safety and the sustainability of healthcare systems [8,9]. Despite these recognized benefits, PBM implementation still faces significant challenges, especially cultural resistance within healthcare institutions where liberal transfusion practices remain entrenched. Traditional didactic strategies may fall short in conveying the complexity of PBM or in promoting long-term behavioral change. In this context, infographics offer a compelling alternative by simplifying technical content and supporting knowledge retention [13].

The visual narratives developed in this study, such as the ‘Surgery Verse’ journey and the ‘Energy Wheel’, reflect a novel application of design thinking in health education. They position PBM not as a collection of isolated interventions, but as an integrated, patient-centered process across the perioperative continuum. By visually aligning patient pathways with the three pillars of PBM, these tools reinforce the concept of coordinated, continuous care.

Incorporating QR codes for supplementary content further enhances interactivity and accessibility. This hybrid model, combining static visualizations with dynamic digital resources, addresses diverse learning styles and supports the ongoing education of healthcare professionals in time-constrained environments.

Framing PBM within the concept of the surgical patient journey is especially relevant. This journey comprises interconnected stages, from preoperative evaluation to postoperative rehabilitation, each requiring coordinated interventions to optimize outcomes. PBM, when integrated into this pathway, enables resource optimization and enhances clinical effectiveness through strategies such as anemia screening and correction, blood conservation during surgery, and structured recovery planning [10]. A fragmented approach may undermine these efforts, while an integrated perspective fosters adherence to evidence-based practice.

Nevertheless, this study has limitations. While the infographics were developed using a robust design methodology and expert input, their effectiveness was not assessed through formal educational interventions or clinical trials. Therefore, their impact on knowledge acquisition, behavioral change, and clinical outcomes remains to be determined. Furthermore, although the development included feedback from PBM-certified professionals, broader usability testing with frontline clinicians and patients would strengthen the generalizability and applicability of the tools.

Future studies should explore the impact of such infographics using both quantitative and qualitative methods, including pre- and post-intervention assessments, focus groups, and implementation science frameworks. Their adaptability across various healthcare settings, cultural contexts, and languages should also be explored to assess scalability.

From a public health perspective, increasing awareness and adherence to PBM is urgent [8,9]. Global blood shortages, exacerbated by the COVID-19 pandemic, underscore the need for sustainable, rational strategies in transfusion medicine. By translating complex evidence into engaging, accessible formats, infographics may function not only as educational tools but also as catalysts for institutional change and policy development.

The methodology described in this study—anchored in user-centered design, iterative development, and multidisciplinary collaboration—offers a replicable framework for other health education topics beyond PBM. Clinical protocols involving complex decision-making, such as perioperative safety or chronic disease management, could benefit from similar infographic-based approaches to improve knowledge transfer and adherence. By emphasizing co-creation with content experts and end-users, and by integrating visual narratives and interactive elements like QR codes, this model can be adapted to diverse educational settings and learner profiles. Its flexibility supports implementation across clinical disciplines and institutional contexts, reinforcing its value as a scalable strategy for translating evidence-based practices into effective, engaging, and actionable learning tools.

## Conclusion

Well-designed infographics can facilitate the translation of complex clinical guidelines, such as those related to PBM, into practical, actionable strategies. They support professional education, enhance multidisciplinary communication, and align with global efforts to improve patient safety and the sustainability of healthcare systems. While further evaluation is warranted, this study offers a replicable model for integrating visual communication into evidence-based practice, particularly in contexts where cognitive overload, time constraints, or educational gaps may hinder effective clinical decision-making.

## Data availability

The data that support the findings of this study are available from the corresponding author upon reasonable request.

## CRediT authorship contribution statement

**Matheus Santos Moitinho:** Conceptualization, Methodology, Investigation, Data curation, Formal analysis, Writing – original draft, Writing – review & editing. **Bianca Meneghini:** Conceptualization, Methodology, Investigation, Data curation, Formal analysis, Writing – original draft, Writing – review & editing. **Rosangela Monteiro:** Conceptualization, Writing – original draft, Writing – review & editing, Supervision. **Anderson Manucci:** Methodology, Investigation, Writing – original draft. **Maria Carolina Guido:** Conceptualization, Writing – original draft, Writing – review & editing. **Guilherme Rabello:** Conceptualization, Writing – original draft, Writing – review & editing, Supervision. **Fabio Biscegli Jatene:** Conceptualization, Supervision, Project administration.

## Conflicts of interest

I declare no conflicts of interest.
